# Selection of appropriate biomarkers to monitor effectiveness of ovarian function suppression in pre-menopausal patients with ER+ breast cancer

**DOI:** 10.1038/s41523-024-00614-w

**Published:** 2024-01-19

**Authors:** Kelly E. McCann, Shari B. Goldfarb, Tiffany A. Traina, Meredith M. Regan, Neelima Vidula, Virginia Kaklamani

**Affiliations:** 1 University of California Los Angeles Medical Center, Los Angeles, CA 90095 USA; 2 Memorial Sloan Kettering Cancer Center, New York, NY 10065 USA; 3 Dana Farber Cancer Institute / Harvard Medical School, Boston, MA 02215 USA; 4 Massachusetts General Hospital, Boston, MA 02114 USA; 5 University of Texas Health Sciences Center San Antonio / MD Anderson Cancer Center, San Antonio, TX 78229 USA

**Keywords:** Predictive markers, Breast cancer

## Abstract

Use of gonadotropin-releasing hormone (GnRH) agonists has been widely adopted to provide reversible ovarian function suppression for pre-menopausal breast cancer patients who are also receiving aromatase inhibitor or tamoxifen therapy based on results of 25 randomized trials representing almost 15,000 women demonstrating a survival benefit with this approach. Past clinical trials designed to establish the efficacy of GnRH agonists have monitored testosterone in the prostate cancer setting and estradiol in the breast cancer setting. We explore the merits of various biomarkers including estradiol, follicle-stimulating hormone (FSH), and luteinizing hormone (LH) and their utility for informing GnRH agonist treatment decisions in breast cancer. Estradiol remains our biomarker of choice in ensuring adequate ovarian function suppression with GnRH agonist therapy among pre-menopausal women with breast cancer. We recommend future trials to continue to focus on estradiol levels as the primary endpoint, as they have in the past.

## Introduction

Approximately 20% of breast cancers in the United States occur in pre-menopausal women^1,2^. Preservation of ovarian function is an important consideration among these young patients who would have otherwise had many viable childbearing years^3^. Ovarian function suppression added to endocrine therapy is one strategy used to prevent premature ovarian insufficiency, impaired fertility, and early menopause while improving the menses recovery rate and potential for pregnancy after chemotherapy^3–5^. Premature ovarian insufficiency was averted among the majority patients ≤ 40 years of age in the Ovarian Protection Trial in Oestrogen Non-responsive Premenopausal Breast Cancer Patients Receiving Adjuvant or Neo-adjuvant Chemotherapy (OPTION) study^6,7^. Successful post-treatment pregnancies have been reported in the Prevention of Menopause Induced by Chemotherapy: a Study in Early Breast Cancer Patients—Gruppo Italiano Mammella 6 (PROMISE-GIM6) and Prevention of Early Menopause Study (POEMS) studies, with no detrimental effects on survival^8,9^. The Pregnancy Outcome and Safety of Interrupting Therapy for Women with Endocrine Responsive Breast Cancer (POSITIVE) trial confirmed that patients attempting pregnancy by temporarily interrupting their endocrine therapy did not increase their risk of breast cancer recurrence in the short-term^10^.

Results from the Suppression of Ovarian Function Trial (SOFT) and Tamoxifen and Exemestane Trial (TEXT) trials, and subsequently the Addition of Ovarian Suppression to Tamoxifen in Young Women With Hormone-Sensitive Breast Cancer Who Remain Premenopausal or Regain Vaginal Bleeding After Chemotherapy (ASTRRA) trial, have indicated that the addition of ovarian function suppression to tamoxifen or an aromatase inhibitor (AI) improves disease-free survival, freedom from distant recurrence, and overall survival^11–13^. A recent meta-analysis of 25 randomized trials using individual patient data from almost 15,000 women confirmed reduction of their 15-year risk of recurrence and death from breast cancer^14^. Consistent 10-year results were found previously in a systematic literature review of 15 studies representing over 11,000 pre-menopausal women with breast cancer^15^. This is particularly true among high-risk patients who remain pre-menopausal after chemotherapy, or patients 40 years and younger who have a higher risk of recurrence^16^. The most substantial benefits were observed when ovarian function suppression is used in combination with an AI, although ovarian function suppression with tamoxifen remains an option, and either of these options conveys a greater benefit than tamoxifen alone in patients with high-risk tumors^16^.

**Fig. 1:**
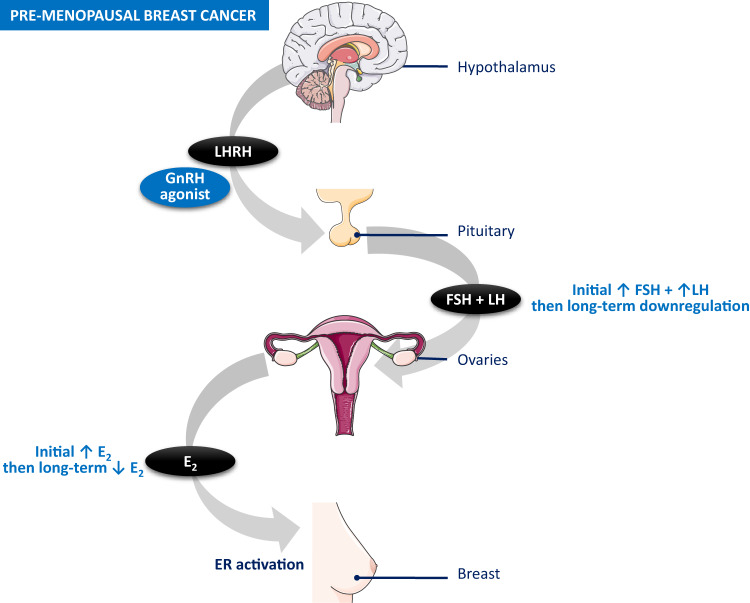
GnRH Agonist Mode of Action in Pre-Menopausal Breast Cancer. (adapted from Lu et al. 2). Upon initiation of GnRH agonist therapy, it mimics endogenous LHRH and stimulates the production of FSH and LH from the pituitary, resulting in an initial surge of E_2_ from the ovaries. With long-term administration of GnRH agonists, the LHRH receptors are downregulated and desensitized, resulting in reduced ovarian hormone production. Abbreviations: GnRH gonadotropin-releasing hormone, LHRH luteinizing hormone-releasing hormone, FSH follicle-stimulating hormone, LH luteinizing hormone, E_2_ estradiol, ER estrogen receptor. Graphics used with permission under a Creative Commons Attribution 3.0 unported license from Servier Medical Art by Servier, available at smart.servier.com.

Ovarian function suppression can be achieved with gonadotropin-releasing hormone (GnRH) agonists, which suppress the release of follicle-stimulating hormone (FSH) and luteinizing hormone (LH) from the pituitary gland by downregulating the GnRH receptors^17^. This reduces the primary method of circulating estradiol production from the ovaries, essentially inducing reversible medical ablation (Fig. 1)^17^. Once GnRH agonist therapy is discontinued and estradiol, FSH, and LH return to normal levels, fertility returns as follicular maturation resumes^17^. The reversibility of medical ovarian function suppression is an advantage over the irreversibility of surgical or radiation ablation, particularly among pre-menopausal women who have the potential for many remaining childbearing years or a desire for future ovarian function^17^. The question arises when designing new studies evaluating GnRH agonists as to which biomarker is the most meaningful surrogate for ovarian function suppression among patients with pre-menopausal estrogen receptor-positive (ER+) breast cancer?

## Utility of biomarkers in pre-menopausal ER+ breast cancer

Approximately 21% to 71% of young women with breast cancer are at risk for early menopause due to chemotherapy^18^. The ovarian function of half of these patients resume within 6 months of completing their chemotherapy^19^. The ovarian recovery can be slower in one of every four patients, with functionality returning 6–24 months after completing their chemotherapy^19^. Eligibility for GnRH agonist therapy in the ASTRRA trial was based on detection of ovarian recovery during 2 years of tamoxifen therapy^19^. The Breast Cancer in Young Women (BCY5) guidelines also support the addition of a GnRH agonist to tamoxifen if ovarian function resumes within 2 years of chemotherapy treatment^20^. Among patients who develop treatment-induced amenorrhea, it has been suggested that biomarkers such as estradiol (E_2_), follicle-stimulating hormone (FSH), and luteinizing hormone (LH) be monitored serially to predict return of ovarian function^21,22^. They can potentially be used to determine whether ovarian function has resumed or if there is a need to initiate ovarian suppression therapy^21,22^. Below, we discuss the merits of each biomarker.

### Estradiol

Estradiol (E_2_) levels ≥ 40 pg/mL are commonly used to establish pre-menopausal status in the absence of regular menstrual cycles for GnRH agonist trial eligibility^23^. Measurements of E_2_ are also useful as a marker for endocrine sensitivity, as well as to monitor compliance/adherence and refine treatment regimens^24,25^. Finding E_2_ levels within the pre-menopausal range in women receiving GnRH agonist treatment is indicative of incomplete ovarian function suppression per the American Society of Clinical Oncology (ASCO) guidelines^26,27^. Typically, an E_2_ level ≤ 10 pg/mL is consistent with menopausal status with ovarian function suppression, and an appropriate level at which an AI can be utilized^28^. Inadequate ovarian function suppression may reduce the impact of AI^28^.

It should be noted that E_2_ monitoring guidelines are not currently available, so the optimal degree of ovarian suppression is undetermined^29^. Although individual clinical laboratories that assay E_2_ levels have established acceptable reference ranges, these may differ between laboratories and may not be consistent between assay methods^30^. The gold standard gas chromatography/mass spectrometry measurement of extracted E_2_ is more sensitive and specific, but is more expensive and tedious to perform than the immunoassay which may cross-react with other steroids since estradiol is not extracted nor purified^22,30–32^. Accuracy and reliability of E_2_ assays are known to falter particularly at the low concentration ranges^29,32,33^. Timing of blood sampling may also affect the results of E_2_ assays, since E_2_ exhibits a diurnal cycle such that early morning peaks may differ from a random sample during the day^34,35^. Although there are no standards regarding timing for estradiol assessment in the real-world setting, the SOFT Estrogen Substudy measured E_2_ at 3, 6, and 12 months^21,36^.

### Follicle-stimulating hormone

Follicle-stimulating hormone is not usually measured in pre-menopausal patients with breast cancer except at baseline to confirm pre-menopausal status and need for ovarian function suppression^37–39^. Certain clinical trials have included FSH as one of the parameters to determine pre-menopausal status, randomization stratification, and/or confirm ovarian ablation^19,40,41^. During GnRH agonist therapy, the secretion of FSH is initially downregulated but progressively returns to baseline levels after 1 month^28^. This is attributed to feedback through inhibin when complete ovarian function suppression is achieved^28^. An exception perhaps is in cases of incomplete ovarian function suppression as in the presence of rising E_2_ levels, which have been reported among women with fibroids^28^. The National Comprehensive Cancer Network^®^ (NCCN^®^) indicates that both E_2_ and FSH levels are used to support the diagnosis of menopause, however clear criteria to guide interpretation of FSH and E_2_ in this population is lacking^42^. Threshold serum E_2_ levels ≥ 40 pg/mL and FSH levels < 30 mIU/mL have been used to detect the resumption of ovarian function in the absence of menses^23^. There is otherwise typically no clinical role of FSH as a stand-alone biomarker in treatment decision-making.

### Luteinizing hormone

In women with breast cancer, LH is sometimes measured at baseline to confirm the patient’s pre-menopausal status and need for ovarian function suppression^37^. Luteinizing hormone is otherwise not followed routinely by oncologists during GnRH agonist therapy as it has no clinical role in treatment decision-making. None of the leading clinical practice guidelines recommend monitoring LH levels in patients with breast cancer, such as ASCO^26,27^, or the International Consensus Symposium for Breast Cancer in Young Women (BCY5) from the European School of Oncology and European Society of Medical Oncology (ESO-ESMO)^20^. Certain trials have included LH assessment as a criteria for eligibility, including the Austrian Breast and Colorectal Cancer Study Group (ABCSG-12) and German Adjuvant Breast Cancer Study Group (GABG) IV-B-93 when LH > 50 pg/mL was used as one of the acceptable parameters to confirm menopause in the absence of regular menstrual cycles^38,39^.

### Other considerations

For a patient with estrogen receptor-positive breast cancer, the goal is estrogen suppression, and therefore it is critical to measure E_2_. However, inadequate estrogen suppression may be due in part to body habitus and elevated body mass index (BMI). Since the primary source of serum estrogen is from adipose tissue, overweight patients are more likely to have larger reserves of estrogen precursors to be metabolized by the aromatase enzyme^43^. Even patients who have had an oophorectomy may have E_2_ levels above post-menopausal range due to contributions from adipose tissue as an exogenous source of estrogen. Patients with high BMI and intact ovaries may be at higher risk of incomplete ovarian function suppression^43^. A subanalysis of the SOFT study indicated that patients with higher BMI (median 27 kg/m^2^) whose E_2_ levels rose above the strict 2.72 pg/mL suboptimal threshold also had lower baseline FSH (median 8 IU/L) and LH (median 7 IU/L) levels^21,24^. Regular E_2_ monitoring has been suggested as the primary biomarker to detect any potential for breakthrough resumption of ovarian function^24^. The threshold for ovarian function suppression is recognized to be E_2_ < 40 pg/mL, although threshold levels as low as 10 pg/mL have been documented^2,28^.

There is less of a concern regarding any potential confounding effects of concomitant therapy such as AI or tamoxifen on E_2_ levels. Since AI primarily inhibits the peripheral conversion of circulating androgens into estrogens (from androstenedione to E_1_, with a minor pathway from testosterone to E_2_), AI will not suppress estrogen to subphysiologic levels if the patient’s ovaries are not being suppressed by other means such as GnRH agonist therapy^44^. Similarly, the mixed agonist/antagonist effect of tamoxifen is thought to be counteracted by the estrogen-depleting effects of goserelin^45^.

## Evaluating GnRH agonist efficacy

The ASCO guidelines on ovarian suppression are based on data from trials involving the 1-month formulation of GnRH agonists^27^. In previous trials designed to evaluate the efficacy of a GnRH agonist, E_2_ < 30 pg/mL was consistently reached indicating ovarian function suppression among the majority of pre-menopausal women^40,46–48^. Estradiol levels were consistent between agents (leuprolide and goserelin) regardless of age, chemotherapy history, taxane regimen, and tumor characteristics^46^. The other biomarkers were less consistent, as differences in FSH levels were observed with patient age (*P* = 0.02), cERB-B2(+) tumors (*P* = 0.05), and lack of taxane in chemotherapy regimen (*P* = 0.05)^46^. Likewise, differences in LH levels were observed between GnRH agonists (*P* = 0.03) and tumor stage (T1 + T2 vs T3 + T4, *P* = 0.03)^46^.

In the prostate cancer setting, neither FSH or LH are measured during GnRH agonist therapy^49^. Testosterone is the biomarker of choice to document castrate levels during androgen deprivation therapy for prostate cancer^49–51^. This correlates to measuring E_2_ in breast cancer. Authorities have released guidance for appropriate clinical endpoints to be used to assess efficacy of GnRH analogs in 2022 for advanced prostate cancer^52^. In this setting, plasma testosterone is suggested as the primary endpoint, with levels of <50 ng/dL defined as the threshold for establishing castrate level^52^. Extrapolating from the prostate cancer setting, it would not be unreasonable to utilize E_2_ as the primary biomarker for GnRH agonist efficacy in breast cancer.

### Considerations for future trials

Based on the above discussion, we recommend that the primary endpoint of ovarian function suppression be measured by E_2_ levels in breast cancer trials. Estradiol should be used since it most closely reflects ovarian function and is the most relevant hormone activating estrogen receptors in the breast (Fig. 1)^2^. The ASCO guidelines indicate that detection of premenopausal E_2_ levels in women while on GnRH agonist therapy is a sign of incomplete ovarian suppression^27^. Measurement of FSH and/or LH levels could be secondary endpoints. Resumption of menses regardless of hormone levels would indicate failure of therapy. The primary concern when evaluating any formulation is the potential for E_2_ escape if the GnRH agonist formulation wears off at the end of the dosing period, before the next dose is scheduled. We suggest trials also checking E_2_ levels towards the end of the dosing interval to confirm it is still suppressed. While in clinical practice it would be important to evaluate for the resumption of menses and/or cyclical fluctuations in climacteric symptoms^27^, whether clinical trial procedures would include such assessments needs to be considered.

## Data Availability

Source data for all figures and tables are provided in the paper. No new data sets have been generated or analyzed for this article.
